# Ethylenediamine-Catalyzed Preparation of Nitrogen-Doped Hierarchically Porous Carbon Aerogel under Hypersaline Condition for High-Performance Supercapacitors and Organic Solvent Absorbents

**DOI:** 10.3390/nano9050771

**Published:** 2019-05-20

**Authors:** Jing Gao, Xuan Zhang, Jiaxing Yang, Junxi Zhou, Mingxing Tong, Qiuyang Jin, Fangna Dai, Guohua Li

**Affiliations:** 1 School of Chemical Engineering, Zhejiang University of Technology, Hangzhou 310032, Zhejiang, China; 2111601105@zjut.edu.cn (X.Z.); 2111701111@zjut.edu.cn (J.Y.); 17816874851@163.com (J.Z.); 2111401029@zjut.edu.cn (M.T.); 2111701069@zjut.edu.cn (Q.J.); 2 School of Materials Science and Engineering, College of Science, China University of Petroleum (East China), Qingdao 266580, Shandong, China; fndai@upc.edu.cn

**Keywords:** carbon aerogel, ethylenediamine, hypersaline condition, supercapacitor, absorbent

## Abstract

The simple and cost-efficient preparation of high-performance nitrogen-doped carbon aerogel (N-CA) for supercapacitors and other applications is still a big challenge. In this work, we have presented a facile strategy to synthesize hierarchically porous N-CA, which is based on solvothermal polymerization of phenol and formaldehyde under hypersaline condition with ethylenediamine (EDA) functioning as both a catalyst and a nitrogen precursor. Benefited from the catalytic effect of EDA on the polymerization, the obtained N-CA has a predominant amount of micropores (micropore ratio: 52%) with large specific surface area (1201.1 m^2^·g^−1^). In addition, nitrogen doping brings N-CA enhanced wettability and reduced electrochemical impedance. Therefore, the N-CA electrode shows high specific capacitance (426 F·g^−1^ at 1 A·g^−1^ in 0.5 M H_2_SO_4_) and excellent cycling stability (104% capacitance retention after 10,000 cycles) in three-electrode systems. Besides, a high energy density of 32.42 Wh·kg^−1^ at 800 W·kg^−1^ can be achieved by symmetric supercapacitor based on the N-CA electrodes, showing its promising application for energy storage. Furthermore, N-CA also exhibits good capacity and long recyclability in the absorption of organic solvents.

## 1. Introduction

Supercapacitors have rapidly developed in recent decades because of their attractive merits such as high power density, short charging–discharging time, long cycling life, and eco-friendly feature, etc., which have triggered significant research efforts into developing electrode materials with high energy and power output and long cycling stability [1,2,3]. Among them, carbon aerogels (CAs) have been considered as promising materials for supercapacitor electrodes due to their advantages such as low density, three-dimensional interconnected network with numerous pores, large surface area, and extraordinary chemical and thermal stability, etc. [4,5]. However, real-world applications of CAs are severely limited. One such reason is that CAs as supercapacitor electrodes only have mediocre electrochemical performances, which is closely related to their poor hydrophily and lack of pseudocapacitance [6]. A well-known strategy to increase the wettability and conductivity of CAs, as well as to provide pseudo-capacitive effects is incorporating heteroatoms (N, S, P etc.) within carbon matrix [7]. For nitrogen doping of CAs, extensive research work has been reported already, in which various materials such as nitrogen-containing commercially available monomers [8,9], nitrogen-containing biomass, waste products, etc., have been widely utilized as starting precursors to synthesize N-doped carbon aerogels (N-CAs) [10,11]. However, most of these precursors only function as nitrogen resources, while having no obvious influences on the pore structure of final products [12,13]. Based on the general view that the presence of narrow micropores is of great importance for improving supercapacitor capacitance [14], it is a great meaningful shortcut to utilize nitrogen precursors to affect the pore structures of N-CAs.

Another reason that hinders the application of CAs is lying in complicated procedures to prepare them, of which a key issue is to maintain their integrity and high porosity during the drying process. A traditional way to solve this issue is evaporating the liquid embedded in gels under supercritical conditions, making the drying process highly dangerous and costly [15]. As a result, some studies have tried using ambient drying to replace the supercritical drying. Unfortunately, these approaches often take very long gelation/activation time and the resulted products generally have large bulk density (>0.2 g·cm^−3^) and small specific surface area (<700 m^2^·g^−1^) due to formation of large cracks during the pyrolysis [16,17]. Recently, a feasible method to prepare highly porous carbonaceous materials under hypersaline conditions has been reported [18,19]. The hypersaline environment, which is created using hygroscopic inorganic salts with Lewis acid feature and strong enough polarizability, is easily fulfilled and represents a promising strategy for N-CA preparation. However, examples about synthesizing N-CAs under hypersaline condition are still rare [9].

Herein, a facile preparation of hierarchically porous N-CAs has been presented. By selecting EDA as both a nitrogen-containing precursor and a catalyst for solvothermal polymerization of phenol and formaldehyde (PF) under hypersaline condition, we have easily obtained N-CA with greatly increased amounts of micropores, improved wettability, and reduced electrochemical impedance as compared to CA. When used as supercapacitor electrodes, the N-CA showed excellent capacitive performances, such as high specific capacitance (426 F·g^−1^ at 1 A·g^−1^), long-time cycling stability (104% capacitance retention after 10,000 cycles at 20 A·g^−1^), and high energy density (32.42 Wh·kg^−1^ at 800 W·kg^−1^), which are superior to those of many other N-CAs reported in the literature. Further investigation indicated that the high capacitive performance of N-CA could be ascribed to the N-doping, which brought benefits including: (i) a large amount of micropores that favor charge accumulation; (ii) a good wettability that facilitates for the infiltration of electrolyte ions to electroactive materials; (iii) a low electrochemical resistance that is beneficial to charge transfer; and (iv) a hierarchically porous structure that enhances the transportation of ions and electrons. Furthermore, the as-prepared N-CA shows high capacity and long recyclability for absorbing organic solvents.

## 2. Materials and Methods

### 2.1. Materials

PTFE was purchased from Macklin Biochemical Co., Ltd. (Shanghai, China). Carbon black and Ethylenediamine were obtained from Aladdin Biochemical Co., Ltd. (Shanghai, China). Phenol was purchased from Shuanglin Chemical Reagent Factory (Hangzhou, China). Formaldehyde was from Lingfeng Chemical Reagent Co., Ltd. (Shanghai, China). N-methylpyrrolidone was obtained from Chemical Reagents Wulian Purchasing and Supply Chemical Plant (Shanghai, China). Zinc chloride, HCl (36.0–38.0%) and H_2_SO_4_ (95.0–98.0%) was purchased from Xilong Scientific Co., Ltd. (Guangdong, China). All other reagents were of analytical purity grade.

### 2.2. Synthesis of Nitrogen-Doped Carbon Aerogels (N-CA)

In a typical synthesis of N-CA, ZnCl_2_ (6.5 g, 47.7 mmol), phenol (0.941 g, 10.0 mmol) and EDA (0.2 mL, 3.0 mmol) were added into formaldehyde (1.5 mL, 40.7 mmol) in an ice-bath to yield a viscous sol. Afterwards, the sol was transferred into a Teflon-lined stainless-steel autoclave (50 mL) and heated at 160 °C for 8 h. The obtained monolith was dried at 135 °C for 12 h in drying oven and subsequently carbonized at 900 °C for 2 h under N_2_ atmosphere (heating rate: 2.5 °C·min^−1^). After the tube furnace was naturally cooled down to room temperature, the black monolith was taken out, immersed in HCl (1 M) for 48 h to remove residual ZnO, washed by deionization (DI) water repeatedly until the pH did not change any more, and then dried in drying oven at 80 °C overnight. The final product was denoted as N-CA.

### 2.3. Synthesis of Carbon Aerogel (CA)

Synthesis of CA was the same as that of the N-CA except that no EDA was used.

### 2.4. Materials Characterization

X-ray diffraction (XRD) data were collected using an X’Pert PRO (PANalytical, Almelo, Netherlands, Cu Kα radiation, λ = 1.5418 Å). Raman and infrared (IR) spectra were recorded on LabRAM HR800 (Horiba JobinYvon, 632.81 nm excitation laser) and Thermo Nicolet Corporation Nicolet 6700, respectively. X-ray photoelectron spectroscopy (XPS) analysis was performed on Kratos AXIS Ultra DLD with Al Kα radiation (45 W). Scanning electron microscope (SEM) and energy dispersive spectrometer (EDS) mapping were carried out on S-4800 (HITACHI, Tokyo, Japan), and transmission electron microscope (TEM) was performed on Tecnai G2 F30 S-Twin (Philips-FEI, Amsterdam, Netherlands). The contact angle was tested on an OCA-20 system (DataPhyisics Instruments GmbH). Nitrogen adsorption/desorption isotherms were recorded on ASAP 2460 (Micromeritics). Before measurements, the sample was degassed under vacuum for 12 h at 180 °C. The Brunauer–Emmett–Teller (BET) model and Density Functional Theory (DFT) method were utilized to obtain values of the specific surface area and curves of pore size distribution, respectively.

### 2.5. Electrochemical Measurements

Cyclic voltammetry (CV), galvanostatic charge−discharge (GCD), and electrochemical impedance spectroscopy (EIS) tests were performed with a CHI660E (China) electrochemical workstation. The electrodes of the supercapacitors were prepared as follows: active materials, polytetrafluoroethylene (PTFE) and carbon black (80:10:10 w/w/w) were mixed in 1-methyl-2-pyrrolidone (NMP) to form slurry. The slurry was then pressed onto a carbon paper (1 × 1 cm^2^) and dried at 80 °C for 12 h to get the electrode. Loading mass of the active material was about 1 mg. For electrochemical tests in three-electrode cell, the counter electrode was platinum and the reference electrode Ag/AgCl. 0.5 M H_2_SO_4_ was used as electrolyte. CV data were collected at 10–300 mV·s^−1^ from −0.25 to 0.75 V. GCD tests were measured at 1–20 A·g^−1^. The gravimetric specific capacitance C_g_ was calculated according to Equation (1):

(1) $$C_{g}=\frac{I\times \Delta t}{m\times \Delta V}$$

where *I* (A), *Δt* (s), *ΔV* (V), and *m* (g) refer to the applied current, discharging time, voltage change, loading mass of the active material, respectively.

For the two-electrode system, a voltage window of 0–1.6 V was applied. The specific energy density (*E*, Wh·kg^−1^) and power density (P, W·kg^−1^) were calculated according to Equations (2)–(4):

(2) $$C_{cell}=\frac{I\times \Delta t}{M\times \Delta V}$$

(3) $$E=\frac{1}{2\times 3.6}C_{cell}{\left(\Delta V\right)}^{2}$$

(4) $$P=\frac{3600\times E}{\Delta t}$$

where *C_cell_* (F·g^−1^) is specific capacitance of the two-electrode cell, and *M* (g) is total loading mass of the active material on the two electrodes.

### 2.6. Absorption of Organic Solvents by the N-CA

The absorption capacity (Q) of the N-CA was evaluated by soaking it in various kinds of organic solvents. For each test, the mass of the N-CA was firstly weighted (*M_i_*, g). After that, the N-CA was immersed in the organic solvent for over 30 min to achieve absorption equilibrium. Then the mass of N-CA was weighted again (*M_f_*, g). The absorption capacity of N-CA was calculated according to Equation (5):

(5) $$Q=\frac{M_{f}-M_{i}}{M_{i}}$$

## 3. Results and Discussion

### 3.1. Fabrication of the N-CA

**Scheme 1:**
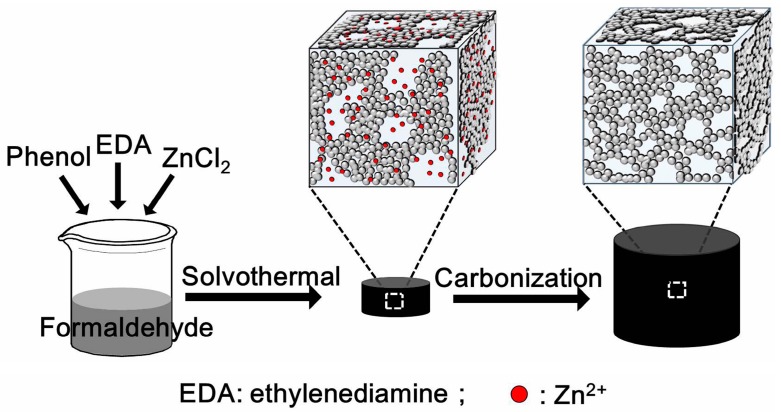
A schematic illustration of the formation of the N-CA.

Scheme 1 illustrates the fabrication process of the N-CA, which was obtained from solvothermal polymerization of PF in the presence of EDA under hypersaline conditions and subsequent carbonization in N_2_ atmosphere. EDA was selected as a catalyst and also a nitrogen precursor according to previously reports that reactions of phenols, formaldehyde and EDA can produce nitrogen containing intermediate polymer frameworks (Scheme S1) [20,21]. The hypersaline condition was provided by ZnCl_2_, which simultaneously functioned as a dehydration agent, foaming agent, and porogen during the preparation process [9,19,22]. A yellowish dark hydrogel was produced after the hydrothermal polymerization, which was then dried under ambient condition, leading to a black monolith. The black monolith showed no large volume shrinkage after the ambient drying, suggesting its porous structure was well kept. Further carbonization of the black monolith in the N_2_ atmosphere resulted in N-CA with greatly expanded volume (Figure S1), confirming the multiple roles of the ZnCl_2_ as mentioned above. The density of N-CA was estimated to be 52.2 mg·cm^−3^, which is comparable to that of some carbon-based aerogels in the reports, such as graphene aerogel (71 mg·cm^−3^) [23] and carbon aerogel derived from winter melon (48 mg·cm^−3^) [24].

### 3.2. Morphology and Structure of the N-CA

**Figure 1:**
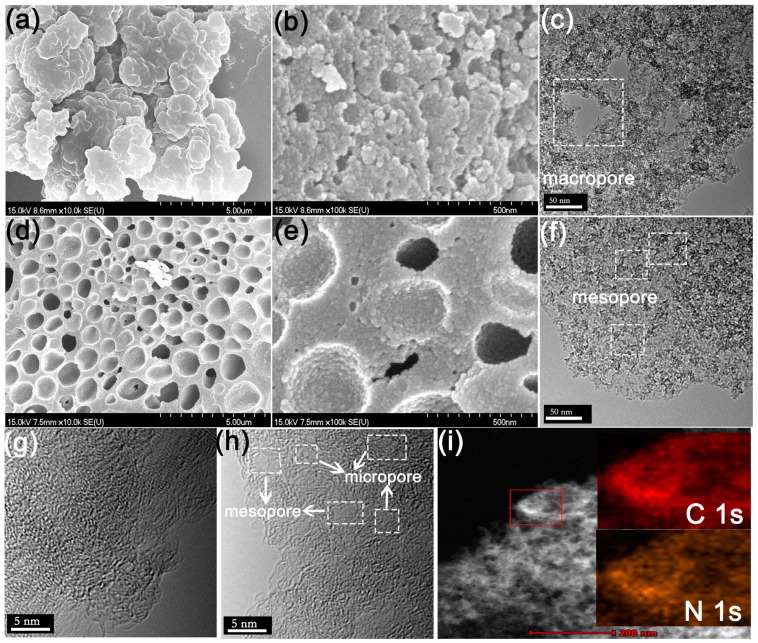
SEM images of the CA (**a**,**b**) and N-CA (**d**,**e**); TEM images of the CA (**c**) and the N-CA (**f**); HRTEM images of the CA (**g**) and the N-CA (**h**); and (**i**) elemental mapping images of the N-CA.

SEM images show that both CA (Figure 1a,b, Figure S2a) and the N-CA (Figure 1d,e, Figure S2b) have a network made up of uniform-sized carbon nanoparticles. The carbon nanoparticles in the CA randomly aggregated together while in the N-CA they orderly assembled into regular bowl-shaped pores with sizes ranging from nanometers to micrometers. Energy-dispersive spectrometry (EDS) element mapping of the N-CA (Figure 1i) obviously shows that the nitrogen element is homogeneously distributed in the N-CA. TEM images show turbostratic graphitic structures for the CA (Figure 1c) and the N-CA (Figure 1f), indicating they are highly amorphous. It is remarkable that interconnected and hierarchical pores can be clearly observed among the carbon nanoparticles from the high resolution transmission electron microscope (HRTEM) images. Compared to the CA that mainly contains macropores (Figure 1g), the N-CA was hierarchically porous with abundant interconnected macropores, mesopores and micropores (Figure 1h), which are beneficial for buffering storage, transportation and electrostatic adsorption of the electrolyte ions, respectively [25]. As a result, the hierarchical pore characteristics of the N-CA are good for electric double layer capacitance (EDLC) applications.

**Figure 2:**
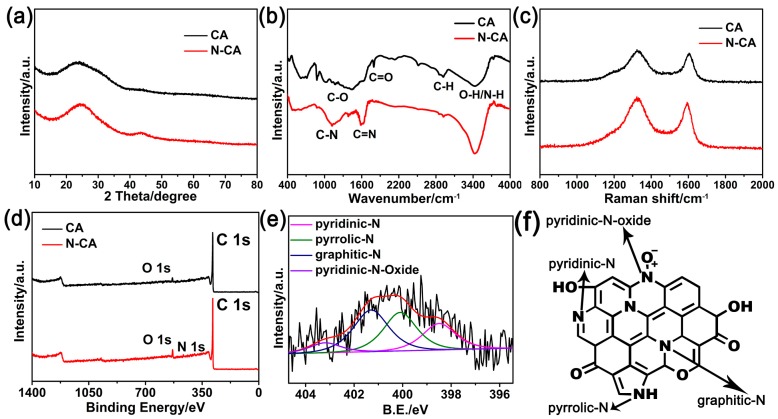
(**a**) XRD patterns, (**b**) IR, and (**c**) Raman spectrum of the CA and the N-CA; (**d**) XPS survey spectra of the CA and the N-CA; (**e**) N 1s high-resolution XPS spectra of the N-CA; and (**f**) schematic model of nitrogen- and oxygen-containing surface functional groups on carbon.

XRD, Raman, and IR spectroscopy were used to study the phase and structures of the as-prepared samples. The XRD patterns of the N-CA presented a wide diffraction peak at 2θ = 23–25° and a weak one at 2θ ≈ 44° (Figure 2a), corresponding to (002) and (100) planes of slightly disordered graphitic carbon, respectively [26]. The IR spectrum of CA showed characteristic stretching vibration peaks of surface –OH (3427 cm^−1^), C−H (2916 cm^−1^), C=O (1793 cm^−1^) and C–O (1178 cm^−1^), suggesting the presence of oxygen-containing functional groups on the surface of the CA (Figure 2b) [27,28]. In contrast, the IR spectrum of N-CA clearly showed new peaks at 1594 cm^−1^ and 1125 cm^−1^ that can be ascribed to N=C and N–C [29]. In addition, the peak at 3427 cm^−1^ in the IR spectra of CA blue-shifted to 3418 cm^−1^ with greatly enhanced intensity in IR spectra of the N-CA (Figure 2b), which might be attributed to stretching vibration of the N−H and the surface –OH [30]. These results clearly confirmed successful introduction of nitrogen heteroatoms into the carbon matrix. Raman spectra of N-CA and CA (Figure 2c) are similar with a D-band and a G-band, which originated from defects in carbon matrix and in-plane stretching vibration of sp^2^-hybridized carbon atoms, respectively [25]. Compared to the G-band of the CA (1605 cm^−1^), a shift of 18 cm^−1^ toward lower energy in the Raman spectrum of the N-CA (1594 cm^−1^) indicated charge transfer from nitrogen atoms to carbon atoms [31]. The intensity ratio of the two bands (I_D_/I_G_) for N-CA (1.11) was higher than that of CA (1.07). Such an enhancement of D band intensity was in consistence with other reports that the N doping often brings in lattice defect in the graphitic carbon matrix [32]. Elemental bonding configurations of the samples were further studied by XPS tests. Survey spectrum of the N-CA showed an additional N 1s peak besides primary C 1s and O 1s peaks (Figure 2d), indicating the successful introduction of nitrogen within the carbon matrix (3.57 wt%). Deconvolution of the N 1s XPS spectrum (Figure 2e) showed four different configurations of N doping, i.e., 398.5, 400.1, 401.3, and 403.2 eV, which correspond to the pyridinic, pyrollic, quaternary, and oxidized N group, respectively (Figure 2f) [32]. The pyridinic N and pyrollic N are beneficial for attracting protons or hydronium ions to the electrode surface due to their electro-rich nature, resulting in pseudocapacitive interactions [33], and the presence of positively charged quaternary N could improve conductivity of carbon materials by facilitating the electron transfer in carbon skeleton [30]. Detailed information about XPS analysis is presented in Figure S3 and Table S1.

**Figure 3:**
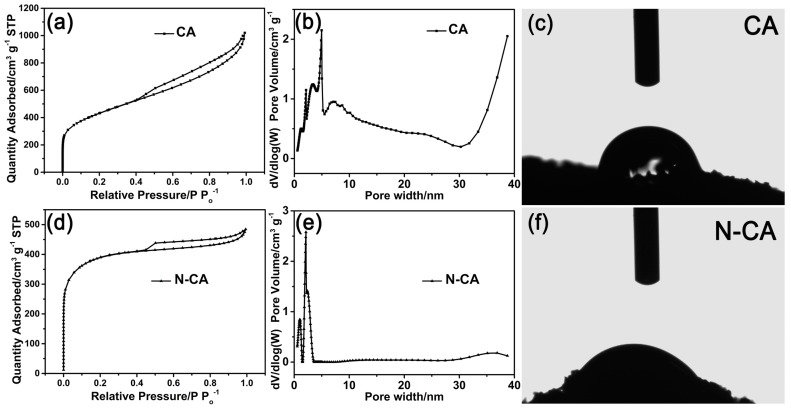
N_2_ adsorption–desorption isotherms of (**a**) CA and (**d**) N-CA; pore size distributions curves of (**b**) CA and (**e**) N-CA; contact angle tests of (**c**) CA and (**d**) N-CA.

Nitrogen adsorption–desorption tests of the samples provided further information about their textile structures, of which the isotherms exhibited combined-features of I/IV type (Figure 3a,d). The steep uptake at P/P_0_ < 0.01 and the hysteresis loop at P/P_0_ = 0.4–1.0 suggest that micropores, mesopores, and macropores are all present in the samples [34], which is in line with the observed results from TEM images. Interestingly, at P/P_0_ from 0.4 to 1.0, the isotherms of CA showed more prominent hysteresis loop and steeper uptakes compared to that of the N-CA, suggesting that CA has fewer micropores [35]. Textural parameters of the samples were summarized in Table 1. A modest decrease of calculated specific surface area (S_BET_) and total pore volume (V_T_) of the N-CA can be observed compared to that of CA, which may be due to the heteroatom doping that has increased additional mass of functionalities in the carbon matrix [36]. Notably, S_micro_ of the N-CA (624.6 m^2^·g^−1^) was 2.7 times larger than that of the CA (229.8 m^2^·g^−1^) and made a contribution of 52% to its S_BET_, revealing that pores in the N-CA were mainly micropores. The pore size distribution (PSD) curves (Figure 3b,e) gave further evidence to this conclusion. Unlike CA that mainly has mesopores with diameters in the range of 2–10 nm, the N-CA presented primary pores with diameters around 1.1, 2.1, and 2.8 nm. The reason might be that CA activated by highly concentrated ZnCl_2_ tends to produce extra large mesopores [22]. However, for N-CA, in addition to influences from the highly concentrated ZnCl_2_, the reaction of phenol, formaldehyde, and EDA produced intermediate polymers doped by nitrogen in atomic scale (Scheme S1), which greatly benefited for producing micropores as nitrogen releasing from the carbon matrix during the carbonization process under high temperature [37]. As a result, the obtained N-CA contains predominant micropores, which is favorable for achieving high S_BET_ and good capacitive performances [32,38]. To investigate if nitrogen doping improved the wettability of N-CA or not, dynamic water contact angle measurements were tested. For the CA, a contact angle of 91.8° suggests it is nearly hydrophobic (Figure 3c). In contrast, the N-CA is quite hydrophilic with a much smaller contact angle of 50.8° (Figure 3f). Such a result clearly shows that the wettability of N-CA is greatly improved. The reason might be that nitrogen doping has brought many C–N bonds, of which the polarity was stronger than that of C–C bonds, leading to improved transport and diffusion of electrolyte ions [38].

### 3.3. Electrochemical Performances of the N-CA

**Figure 4:**
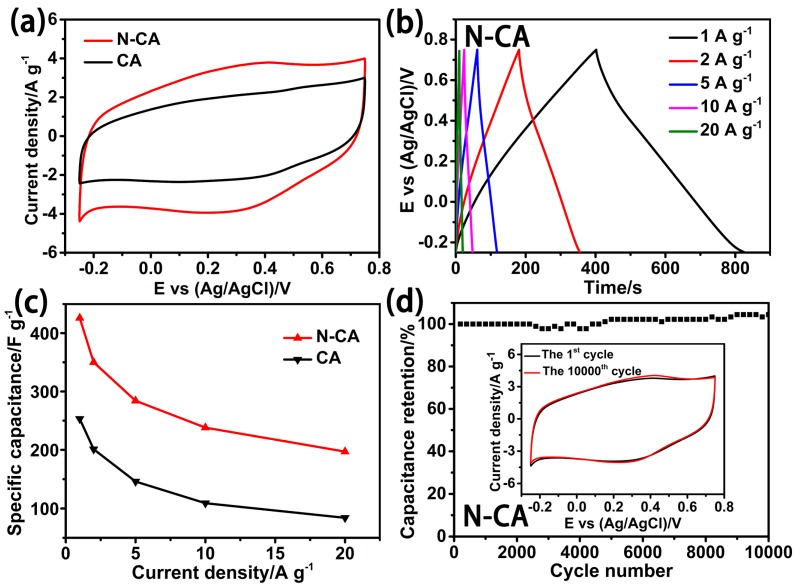
(**a**) CV curves of CA and N-CA at a scan rate of 10 mV·s^−1^; (**b**) GCD curves of the N-CA at various current densities; (**c**) gravimetric capacitances of the N-CA and the CA at different current densities; and (**d**) cycling stability of the N-CA at 20 A·g^−1^, inset: CV curves of the N-CA electrode at the 1st (black) and the 10,000th cycle (red) at 10 mV·s^−1^, respectively.

To explore the electrochemical performances of the N-CA, cyclic voltammetry (CV), and galvanostatic charge–discharge (GCD) tests have been performed. Figure 4a illustrates CV curves of the CA and N-CA electrodes at 10 mV·s^−1^. The CV curves had quasi-rectangular shapes with obvious redox peaks at 0.2–0.5 V, indicating both EDLC and pseudocapacitance properties [39]. It is worthy to note that the N-CA had much larger double-layer currents and exhibited more prominent redox peaks at 0.2–0.5 V than the CA, suggesting it had larger capacitance. CV curves of the N-CA still had nearly rectangular shapes even at 300 mV·s^−1^ (Figure S4c), indicating quick charge propagation capacity. Figure 4b shows the GCD profiles of N-CA, which are nearly triangular with some deviations from linear characteristics, indicating the presence of redox reactions during the charge–discharge process. Besides, the small voltage drop even at high current loads suggests that the N-CA electrode had low internal resistance, which is beneficial for large power output in practical applications [40]. Calculated gravimetric specific capacitance (C_g_) of the N-CA electrode is 426 F·g^−1^ at 1 A·g^−1^. This value was much higher than that of the CA electrode (253 A·g^−1^) and many reported nitrogen-doping carbonaceous materials (Table 2). With smaller S_BET_ and larger C_g_, the N-CA electrode showed an area-normalized capacitance 2.1 times higher than that of the CA (35.5 μF·cm^−2^ versus 17.1 μF·cm^−2^ at 1 A·g^−1^). Rate capacity of the N-CA electrode was further investigated (Figure 4c). C_g_ gradually decreased as the current density increased, which may be related to factors such as limited amount of mesopores, increased internal ohmic resistance under high current loads, and interactions between surface functionalities and electrolyte ions and others [39]. Figure 4d shows cycle stability of the N-CA sample, which was tested by continuous GCD at 20 A·g^−1^ for 10,000 cycles. A capacitance retention of 104% could be achieved after 10,000 cycles. Moreover, CV curves of the N-CA after the 1st cycle and the 10,000th cycle were nearly identical, showing that the N-CA electrode has excellent long-term cycle stability.

**Figure 5:**
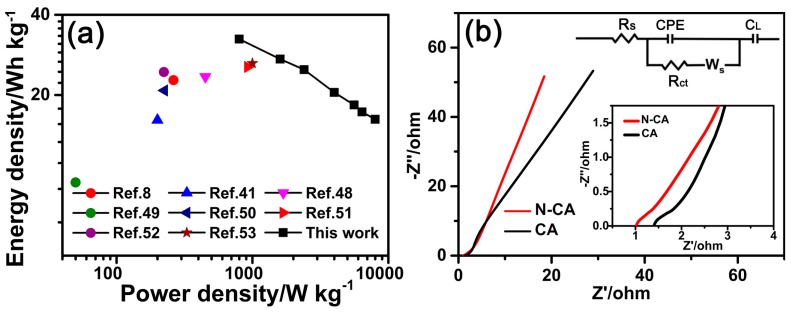
(**a**) Ragone plot of N-CA; (**b**) EIS tests of the CA and the N-CA. Upper inset: the electrical equivalent circuit that includes R_s_, R_ct_, a Warburg element W_o_, a pseudocapacitive element C_L_ from redox processes and a constant phase element (CPE) accounting for EDLC; lower inset: enlarged view of the Nyquist plots.

To evaluate practical capacitive performances of the electrode materials, a symmetric two-electrode cell based on the N-CA electrodes was employed. CV, GCD profiles, and Ragone plot of the cell are shown in Figure S5 and Figure 5a, respectively. Strikingly, for the N-CA, a high energy density of 32.42 Wh·kg^−1^ was achieved at 800 W·kg^−1^. Moreover, it was maintained at 9.33 Wh·kg^−1^ even at a high power density of 24 kW·kg^−1^. Such performances are superior to those of many reported N-doped carbon materials in two-electrode systems (Figure 5a and Table S2) [8,41,48,49,50,51,52,53]. The high energy density of the N-CA can be ascribed to both its high C_g_ and a wide potential window of 1.6 V (Figure S5a) in the H_2_SO_4_ aqueous electrolyte [34].

To further explore reasons why the N-CA showed much-enhanced electrochemical performances compared to those of CA, the fundamental transport behavior of these electrode materials was then examined by electrochemical impedance spectroscopy (EIS). Figure 5b presents the Nyquist plots, which was measured at 100 kHz–0.01 Hz with an ac perturbation of 5 mV. EIS results are fitted using Zview software and the equivalent circuits are given as inset of Figure 5b. At high frequencies, the intercept on Z’ (real impedance) axis represents the bulky solution resistance (R_s_) of the electrodes, which is produced from the electrolyte and contact between current collector and the electrodes. The Nyquist plot of N-CA showed smaller R_s_ than that of CA (1.01 Ω vs. 1.40 Ω), which could be ascribed to its enhanced wettability and highly interconnected structure with hierarchical porosity. At high-medium frequencies, both plots had semicircles that were representative of the charge-transfer resistance (R_ct_) [54]. It should be noted that the semicircle on the Nyquist plot of the N-CA was smaller than that of CA, suggesting there were more accumulated electrolyte ions and redox reactions of heteroatoms on the surface of the N-CA electrode [25]. The simulated value of the R_ct_ provided further evidence to this conclusion, which was as small as 0.207 Ω for the N-CA electrode. At low frequency, the Nyquist plots were linear with different slopes, reflecting diffusion behavior of electrolyte ions within the electrode. Compared to that of CA, a steeper slope of the plot for the N-CA electrode indicated it is more representative of an ideal capacitor [55]. The reason may be that nitrogen doping could improve conductivity and wettability of the N-CA and the 3D interconnected structure of the N-CA with properly distributed hierarchical pores could reduce transport resistance and shorten diffusion distance of electrolyte ions [56].

### 3.4. Absorption Capacity of the N-CA

**Figure 6:**
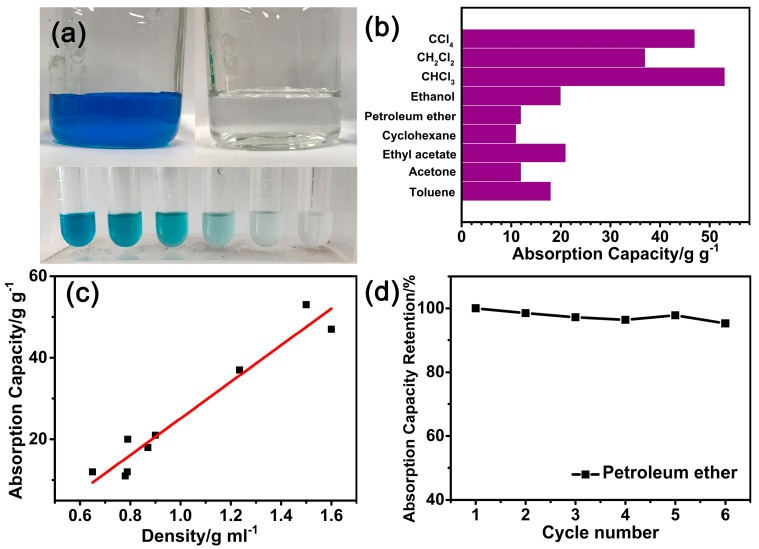
(**a**) Images showing absorption process of MB by N-CA within 2 h; (**b**) absorption capacities of N-CA for some organic solvents; (**c**) absorption capacities of the N-CA measured for investigated organic solvents in terms of the solvent densities; and (**d**) recyclability of the N-CA for absorption of petroleum ether after six heating cycles.

Carbon materials have been frequently reported for their high absorption capacity [56]. As a result, the as-prepared N-CA was also measured to absorb various organic solvents and dye (Figure 6). Figure 6a shows the absorption of methylene blue (MB, 0.2 mg) by the N-CA. When the N-CA was brought to contact with the MB, it adsorbed the MB completely within 2 h. Other studied organic solvents are common pollutants in industry and our daily life, as presented in Figure 6b. In general, the capacity of the N-CA to absorb these liquids was 12–53 times to its own weight. Figure 6c reveals that the absorption capacity had nearly linear relationship with the density of the organic solvents, indicating that the N-CA has a quite stable storing volume. In addition, by choosing petroleum ether as an example, we tested the recyclability of N-CA. Since N-CA has a robust structure, the absorbed petroleum ether was retrieved by drying at 100 °C, which is higher than its boiling point, after which the residual mass was recorded. Such a process was repeated for six times. It can be observed from Figure 6d that the absorption capacity after six cycles was 95.3% to its initial value, indicating that the N-CA has good recycling performance with stable reusability.

## 4. Conclusions

A facile method for fabricating hierarchically porous nitrogen-doped carbon aerogel using PF and EDA as precursors has been developed. The solvothermal polymerization of PF under hypersaline conditions largely shortened the production period and simplified the synthetic steps, and the catalytic effect of EDA on the polymerization of PF not only achieved the nitrogen doping, but also greatly optimized the pore structures of final products, leading to N-CA with predominant micropores. Benefited from the nitrogen-doping, the N-CA showed enhanced wettability and reduced electrochemical impedance, making it exhibit high specific capacitance, long cycling life, and high energy density. In addition, the N-CA shows high capacity and good recyclability in absorbing organic solvents. Our facile approach to synthesize the hierarchical porous N-CA may provide new avenues for developing promising materials for high-performance supercapacitors.

## Figures and Tables

**Table 1:** Textural parameters of CA and N-CA.

Samples	S_BET_^1^/m^2^·g^−1^	V_T_^2^/cm^3^·g^−1^	S_micro_^3^/m^2^·g^−1^	V_micro_^4^/cm^3^·g^−1^	S_E_^5^/m^2^·g^−1^	D ^6^/nm
CA	1479.1	1.50	229.8	0.12	1049.92	5.0
N-CA	1201.1	0.75	624.6	0.35	576.5	3.7

^1^ BET surface area. ^2^ Total pore volume at (P/P_0_) = 0.993. ^3^ t-Plot micropore area. ^4^ t-Plot micropore volume. ^5^ t-Plot external surface area. ^6^ average pore width.

**Table 2:** C_g_ of previously reported carbon-based electrode materials and the N-CA.

Items	C_g_^1^	I ^2^	Electrolyte	Ref.
N-enriched porous carbon spheres	388	1	1M H_2_SO_4_	[20]
N-doped carbon fiber aerogel	365	0.5	6M KOH	[41]
N-doped carbon nanofiber paper	209	1	1M KOH	[42]
B/N co-doped carbon	304	0.1	1M H_2_SO_4_	[36]
N-doped porous carbon	238	0.5	6M KOH	[43]
N/S co-doped carbon	322	1	6M KOH	[44]
N-doped porous carbon nanofibers	245	1	6M KOH	[45]
N/P co-doped graphene	219	0.25	6M KOH	[46]
N/S co-doped porous carbon	302	1	6M KOH	[47]
N-doped carbon aerogel	426	1	0.5M H_2_SO_4_	This work

^1^ unit is F·g^−1^; ^2^ unit is A·g^−1^.

## Supplementary Materials

The following are available online at https://www.mdpi.com/2079-4991/9/5/771/s1, Scheme S1. Representation of possible phenol–formaldehyde–ethylenediamine polymerization. Four and five are possible intermediate polymer frameworks. Figure S1. Photo images of N-CA before (a) and after (b) carbonization. Figure S2. SEM images of CA (a) and N-CA (b). Figure S3. (a) C 1s and (b) O 1s high-resolution XPS spectra of CA; (c) C 1s and (d) O 1s high-resolution XPS spectra of the N-CA. Figure S4. (a) CV curves of the CA at 10–300 mV s^−1^; (b) GCD curves of the CA at 1–20 A g^−1^; and (c) CV curves of the N-CA at 10–300 mV s^−1^. Figure S5. Electrochemical properties tested in a two-electrode cell. (a) CV curves of the cell at 10–300 mV s^−1^; (b) GCD curves of the cell at 1–10 A g^−1^; and (c) gravimetric capacitances of the cell at different charge–discharge current densities. Table S1. Deconvolution results of N 1s, C 1s, and O 1s of N-CA and CA. Table S2. Ragone data of various carbon materials. Table S3. Comparing the adsorption capacity of carbon materials in the literature.
