# Supplementary material for: Ethylenediamine-Catalyzed Preparation of Nitrogen-Doped Hierarchically Porous Carbon Aerogel under Hypersaline Condition for High-Performance Supercapacitors and Organic Solvent Absorbents
Source: Nanomaterials (Basel). 2019 May 20;9(5):771. doi: 10.3390/nano9050771 (PMC6566518; doi:10.3390/nano9050771)
Supplement: Supplementary file 1 [file nanomaterials-09-00771-s001.pdf]

Supplementary material

Ethylenediamine-Catalyzed Preparation of Nitrogen-Doped Hierarchically Porous Carbon Aerogel under Hypersaline Condition for High-Performance Supercapacitors and Organic Solvent Absorbents

Jing Gao [1,*], Xuan Zhang [1], Jiaxing Yang [1], Junxi Zhou [1], Mingxing Tong [1], Qiuyang Jin [1], Fangna Dai [2] and Guohua Li [1,*]

1. School of Chemical Engineering, Zhejiang University of Technology, Hangzhou, Zhejiang, 310032, P.R. China; 2111601105@zjut.edu.cn (X.Z.); 2111701111@zjut.edu.cn (J.Y.); 17816874851@163.com (J.Z.); 2111401029@zjut.edu.cn (M.T.); 2111701069@zjut.edu.cn (Q.J.);
2. School of Materials Science and Engineering, College of Science, China University of Petroleum (East China), Qingdao, Shandong 266580, P.R. China; fndai@upc.edu.cn

* Correspondence: happysmilejing0606@aliyun.com (J.G.); nanozjut@zjut.edu.cn (G.L.)
Tel.: (+86)-571-88320521(G.L.)

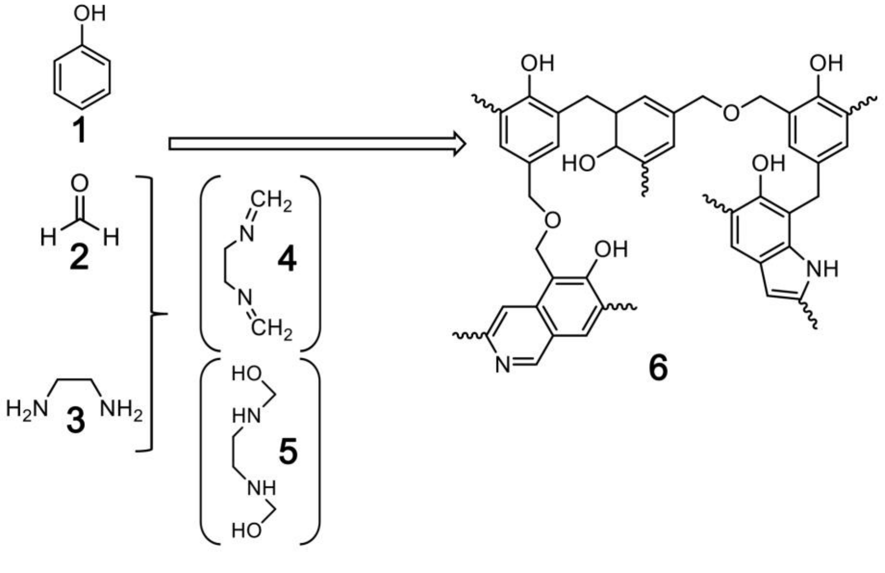

Scheme S1. Representation of possible phenol-formaldehyde-ethylenediamine polymerization. 4, 5 are possible intermediate polymer frameworks.

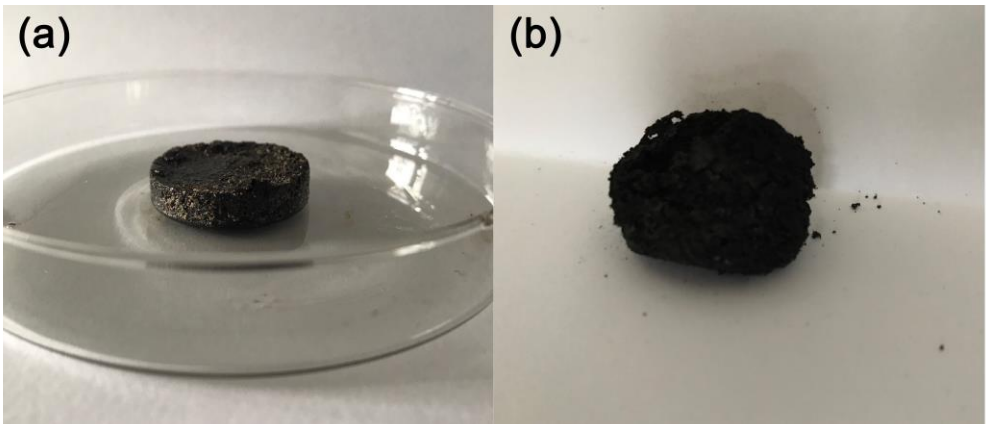

Figure S1. Photo images of the N-CA before (a) and after (b) carbonization.

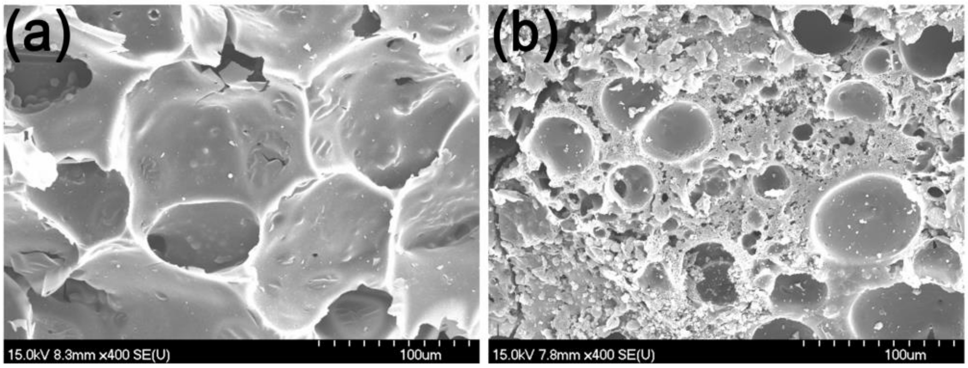

Figure S2. SEM images of the CA (a) and the N-CA (b).

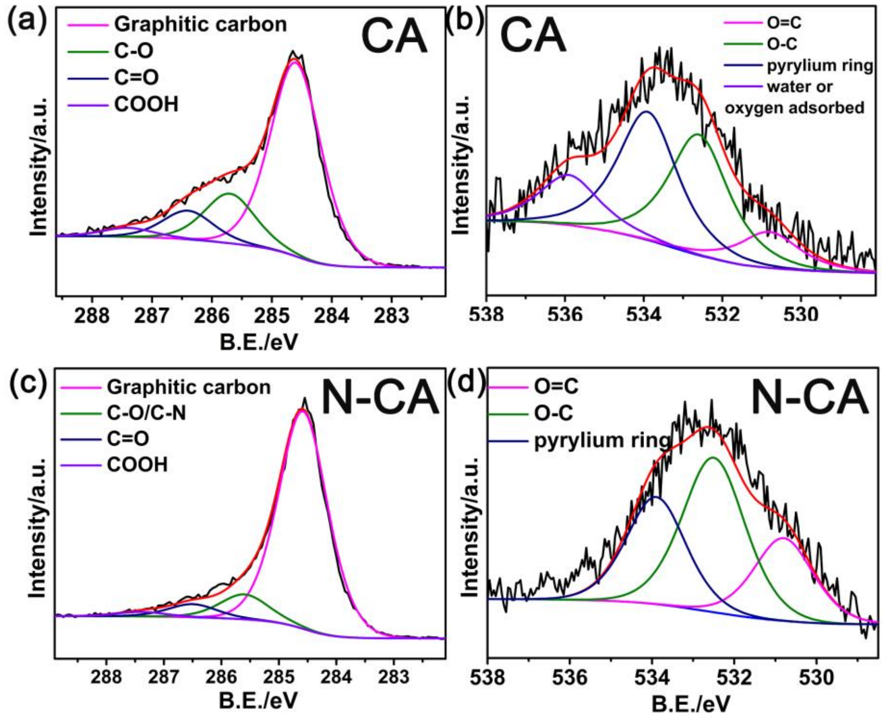

Figure S3. (a) C 1s and (b) O 1s high-resolution XPS spectra of the CA; (c) C 1s and (d) O 1s high-resolution XPS spectra of the N-CA.

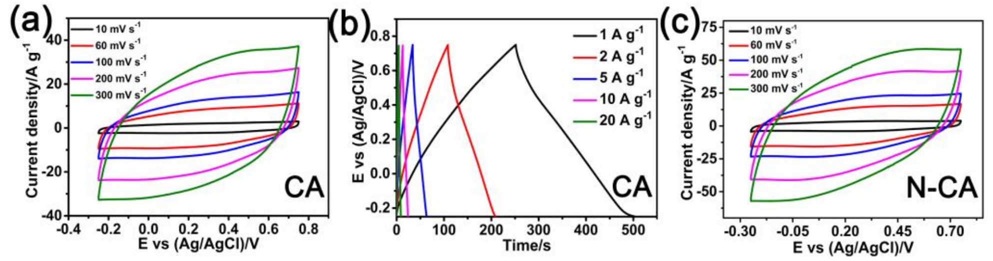

Figure S4. (a) CV curves of the CA at 10-300 mV s^{-1}; (b) GCD curves of the CA at 1-20 A g^{-1}; (c) CV curves of the N-CA at 10-300 mV s^{-1}.

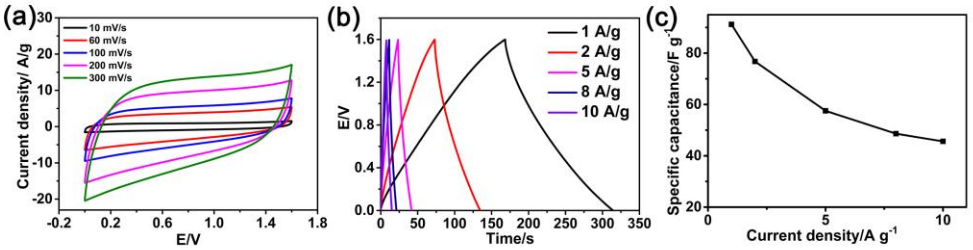

Figure S5. Electrochemical properties tested in a two-electrode cell. (a) CV curves of the cell at 10-300 mV s^{-1}; (b) GCD curves of the cell at 1-10 A g^{-1}; (c) Gravimetric capacitances of the cell at different charge–discharge current densities.

Table S1. Deconvolution results of N 1s, C 1s and O 1s of N-CA and CA.

	Deconvolution			
C 1s	Graphitic carbon	C-O/C-N	C=O	O=C-O
	284.6	285.6+0.1	286.5-0.1	287.4
O 1s	O=C	O-C	Pyrylium [1] ring	Chemisorbed oxygen
	530.8	532.5+0.1	533.9	535.9

Table S2. Ragone data of various carbon materials.

Items	Electrolyte	Energy density /Wh kg^{-1}	Power density /W kg^{-1}	Ref.
N-doped mesoporous carbon	6 M KOH	8.6	0.1[a]	[2]
N-doped carbon plate	6 M KOH	11.05	250	[3]
N-doped carbon scaffold	6 M KOH	11.7	1413	[4]
N-doped carbon networks	0.5 M Na_2SO_4	15.8	450	[5]
N-doped worm-like carbon	1 M Na_2SO_4	23.43	450	[6]
N-doped carbon nanosheets	0.5 M Na_2SO_4	20.8	225	[7]
O/N-co-doped porous carbons	1 M Na_2SO_4	25.5	923	[8]
N-doped carbon aerogels	1 M H_2SO_4	22.75	262.5	[9]
carbon nanoflakes	1 M Na_2SO_4	24.4	223	[10]
Microporous carbon from PMF resin	6 M KOH	26.3	1000	[11]
Biomass-derived Carbon	1 M Na_2SO_4	37.3	149	[12]
Porous carbons	1 M Na_2SO_4	37.29	160	[13]
N-doped carbon fiber aerogel	6 M KOH	16.1	200	[14]
N-doped carbon/graphene aerogel	Polyvinyl alcohol/H_2SO_4	12.4	0.2[a]	[15]
N-doped porous carbon	6 M KOH	9.4	50	[16]
N-doped carbon aerogel	2 M H_2SO_4	31.25	250	[17]
N, P-codoped graphene	6 M KOH	8.2	162	[18]
N-doped carbon aerogel	0.5 M H_2SO_4	32.42	800	This work

[a] unit is A g^{-1}

Table S3. Comparison of the adsorption capacity of carbon materials in the literature.

Items	Adsorption capacity /g g^{-1}	Ref.
Macroporous carbon materials	32-77	[19]
Carbon aerogel	16-50	[20]
Carbon nanotubes	3.46-11.90	[21]
N-doped graphene aerogel	13-32	[22]
Carbon fiber aerogel	22-50	[23]
Macro/mesoporous carbon	23-48	[24]
Macro/mesoporous carbon monoliths	20	[25]
Carbon material	81–171	[26]
Carbon aerogel	14-26	[27]
CNF/MWCNT carbon aerogel	110	[28]
Carbon aerogel	10-20	[29]
Graphene aerogel	44-68	[30]
Carbon aerogel	35-45	[31]
N-doped carbon aerogel	12-53	This work

References

1. Far, H.M.; Donthula, S.; Taghvaee, T.; Saeed, A.M.; Garr, Z.; Sotiriou-Leventis, C., Leventis, N. Air-oxidation of phenolic resin aerogels: backbone reorganization, formation of ring-fused pyrylium cations, and the effect on microporous carbons with enhanced surface areas. *RSC Adv.* **2017**, *7*, 51104-51120.
2. Li, M.; Xue, J.M. Integrated synthesis of nitrogen-doped mesoporous carbon from melamine resins with superior performance in supercapacitors. *J. Phys. Chem. C* **2014**, *118*, 2507-2517.
3. Dong, Y.H.; Wang, W.X.; Quan, H.Y.; Huang, Z.N.; Chen, D.Z., Guo, L. Nitrogen-doped foam-like carbon plate consisting of carbon tubes as high-performance electrode materials for supercapacitors. *ChemElectroChem* **2016**, *3*, 814-821.
4. Xu, L.L.; Yin, D.D.; Zhao, H.Y.; Li, N.; Chen, S.H.; Xia, J.L.; Lu, B.A., Du, Y.P. Carbon thin film wrapped around a three-dimensional nitrogen-doped carbon scaffold for superior-performance supercapacitors. *Chem. Eur. J.* **2017**, *23*, 9641-9646.
5. Peng, H.; Ma, G.F.; Sun, K.J.; Mu, J.J.; Zhang, Z., Lei, Z.Q. Facile synthesis of poly(p-phenylenediamine)-derived three-dimensional porous nitrogen-doped carbon networks for high performance supercapacitors. *J. Phys. Chem. C* **2014**, *118*, 29507-29516.
6. Sun, G.L.; Ma, L.Y.; Ran, J.B.; Li, B.; Shen, X.Y., Tong, H. Templated synthesis and activation of highly nitrogen-doped worm-like carbon composites based on melamine-urea-formaldehyde resins for high performance supercapacitors. *Electrochim. Acta* **2016**, *194*, 168-178.
7. Peng, H.; Ma, G.F.; Sun, K.J.; Mu, J.J., Lei, Z.Q. One-step preparation of ultrathin nitrogen-doped carbon nanosheets with ultrahigh pore volume for high-performance supercapacitors. *J. Mater. Chem. A* **2014**, *2*, 17297-17301.
8. Tang, C.G.; Liu, Y.J.; Yang, D.G.; Yang, M., Li, H.M. Oxygen and nitrogen co-doped porous carbons with finely-layered schistose structure for high-rate-performance supercapacitors. *Carbon* **2017**, *122*, 538-546.
9. Wei, X.J.; Wan, S.G., Gao, S.Y. Self-assembly-template engineering nitrogen-doped carbon aerogels for high-rate supercapacitors. *Nano Energy* **2016**, *28*, 206-215.

10. Mao, N.; Wang, H.L.; Sui, Y.; Cui, Y.P.; Pokrzywinski, J.; Shi, J.; Liu , W.; Chen, S.G.; Wang, X., Mitlin, D. Extremely high-rate aqueous supercapacitor fabricated using doped carbon nanoflakes with large surface area and mesopores at near-commercial mass loading. *Nano Res.* **2017**, *10*, 1767-1783.
11. Xiang, X.X.; Liu , E.H.; Xie , H.; Tian, Y.Y.; Wu, Y.H.; Wu, Z.L., Zhu, Y.H. Highly stable performance of supercapacitors using microporous carbon derive from phenol-melamine-formaldehyde resin. *J. Solid State Electrochem.* **2012**, *16*, 2661-2666.
12. Shan, D.D.; Yang, J.; Liu, W.; Yan, J., Fan, Z.J. Biomass-derived three-dimensional honeycomb-like hierarchical structured carbon for ultrahigh energy density asymmetric supercapacitors. *J. Mater. Chem. A* **2016**, *4*, 13589-13602.
13. Su, X.L.; Jiang, S.; Zheng, G.P.; Zheng, X.C.; Yang, J.H., Liu, Z.Y. High-performance supercapacitors based on porous activated carbons from cattail wool. *J. Mater. Sci.* **2018**, *53*, 9191-9205.
14. Du, J.; Liu, L.; Hu, Z.P.; Yu, Y.F.; Zhang, Y.; Hou, S.L., Chen, A.B. Raw-cotton-derived N-doped carbon fiber aerogel as an efficient electrode for electrochemical capacitors. *ACS Sustainable Chem. Eng.* **2018**, *6*, 4008-4015.
15. Wang, C.; Huang, Y.S.; Pan, H.; Jiang, J.Z.; Yang, X.W.; Xu, Z.X.; Tian, H.; Han, S., Wu, D.Q. Nitrogen-doped porous carbon/graphene aerogel with much enhanced capacitive behaviors. *Electrochim. Acta* **2016**, *215*, 100-107.
16. Li, B.Q.; Cheng, Y.F.; Dong, L.P.; Wang, Y.M.; Chen, J.C.; Huang, C.F.; Wei, D.Q.; Feng, Y.j.; Jia, D.C., Zhou, Y. Nitrogen doped and hierarchically porous carbons derived from chitosan hydrogel via rapid microwave carbonization for high-performance supercapacitors. *Carbon* **2017**, *122*, 592-603.
17. Zhang, X.F.; Zhao, J.Q.; He, X.; Li, Q.Y.; Ao, C.H.; Xia, T.; Zhang, W.; Lu, C.H., Deng, Y.L. Mechanically robust and highly compressible electrochemical supercapacitors from nitrogen-doped carbon aerogels. *Carbon* **2018**, *127*, 236-244.
18. Xia, K.S.; Huang, Z.Y.; Zheng, L.; Han, B.; Gao, Q.; Zhou, C.G.; Wang , H.Q., Wu, J.P. Facile and controllable synthesis of N/P co-doped graphene for high-performance supercapacitors. *J. Power Sources* **2017**, *365*, 380-388.
19. Wang, B.; Karthikeyan, R.; Lu, X.Y.; Xuan, J., Leung, M.K.H. Hollow carbon fibers derived from natural cotton as effective sorbents for oil spill cleanup. *Ind. Eng. Chem. Res.* **2013**, *52*, 18251-18261.
20. Li, Y.Q.; Samad, Y.A.; Polychronopoulou, K.; Alhassan, S.M., Liao, K. Carbon aerogel from winter melon for highly efficient and recyclable oils and organic solvents absorption. *ACS Sustainable Chem. Eng.* **2014**, *2*, 1492-1497.
21. Sun, H.X.; La, P.Q.; Zhu, Z.Q.; Liang, W.D.; Yang, B.P.; Zhao, X.H.; Pei, C.J., Li, A. Hydrophobic carbon nanotubes for removal of oils and organics from water. *J. Mater. Sci.* **2014**, *49*, 6855-6861.
22. Du, Y.X.; Liu, L.B.; Xiang, Y., Zhang, Q. Enhanced electrochemical capacitance and oil-absorbability of N-doped graphene aerogel by using amino-functionalized silica as template and doping agent. *J. Power Sources* **2018**, *379*, 240-248.
23. Jiao, Y.; Wan, C.C., Li, J. Synthesis of carbon fiber aerogel from natural bamboo fiber and its application as a green high-efficiency and recyclable adsorbent. *Mater. Design* **2016**, *107*, 26-32.
24. Tao, G.J.; Zhang, L.X.; Hua, Z.L.; Chen, Y.; Guo, L.M.; Zhang, J.M.; Shu, Z.; Gao, J.H.; Chen, H.R.; Wu, W.; Liu, Z.W., Shi, J.L. Highly efficient adsorbents based on hierarchically macro/mesoporous carbon monoliths with strong hydrophobicity. *Carbon* **2014**, *66*, 547-559.
25. Hu, W.J.H.; Zhang, P.B.; Liu, X.K.; Yan, B.; Xiang, L.; Zhang, J.W.; Gong, L.; Huang, J.; Cui, K.X.; Zhu, L.P., Zeng, H.B. An amphiphobic graphene-based hydrogel as oil-water separator and oil fence material. *Chem. Eng. J.* **2018**, *353*, 708-716.

26. Gao, S.Y.; Li, X.G.; Li, L.Y., Wei, X.J. A versatile biomass derived carbon material for oxygen reduction reaction, supercapacitors and oil/water separation. *Nano Energy* **2017**, *33*, 334-342.
27. E, L.; Li, W.; Ma, C.H., Liu, S.X. An ultra-lightweight recyclable carbon aerogel from bleached softwood kraft pulp for efficient oil and organic absorption. *Mater. Chem. Phys.* **2018**, *214*, 291-296.
28. Xu, Z.Y.; Jiang, X.D.; Tan, S.C.; Wu, W.B.; Shi, J.T.; Zhou, H., Chen, P. Preparation and characterisation of CNF/MWCNT carbon aerogel as efficient adsorbents. *IFT Nanobiotechnol* **2018**, *12*, 500-504.
29. Yu, M.; Han, Y.Y.; Li, J., Wang, L.J. Magnetic carbon aerogel pyrolysis from sodium carboxymethyl cellulose/sodium montmorillonite composite aerogel for removal of organic contamination. *J. Porous Mater.* **2017**, *25*, 657-664.
30. Riaz, M.A.; Hadi, P.; Abidi, I.H.; Tyagi, A.; Ou, X.W., Luo, Z.T. Recyclable 3D graphene aerogel with bimodal pore structure for ultrafast and selective oil sorption from water. *RSC Adv.* **2017**, *7*, 29722-29731.
31. Yin, A.S.; Xu, F., Zhang, X.M. Fabrication of biomass-derived carbon aerogels with high adsorption of oils and organic solvents: effect of hydrothermal and post-pyrolysis processes. *Materials* **2016**, *9*, 1-9.
